# Organic fertilizer amendments reshape soil microbial communities and metabolic functions in sweet cherry production

**DOI:** 10.3389/fpls.2026.1920116

**Published:** 2026-09-29

**Authors:** Xue‐feng Jiang, Yong Pang, Kuan-Ying Li, Zong-Xin Dou, Jing Ren

**Affiliations:** 1 Institute of Forestry, Fruit and Flower Research, Gansu Academy of Agricultural Sciences, Lanzhou, China; 2 Gansu Vocational College of Agriculture, Lanzhou, China

**Keywords:** correlation analysis, EM inoculant, metabolomics, microbial community, organic substrate, *Prunus avium L*., rhizosphere microecology

## Abstract

**Introduction:**

Soil microorganism and metabolite-mediated root-soil interactions play a pivotal role in regulating fertilizer utilization efficiency and sweet cherry growth.

**Methods:**

Four treatment groups were established: a control group (CK, garden soil with conventional fertilization) and three amended groups (T1: sheep manure-biochar-EM inoculant; T2: chicken manure-biochar-EM inoculant; T3: biochar-EM inoculant).

**Results and discussion:**

Soil physicochemical assays indicated fertilization boosted available N, P, K and organic matter but reduced pH, with T3 yielding the greatest nutrient accumulation. By integrating high-throughput sequencing and LC-MS-based non-targeted metabolomics, we characterized the structure and diversity of root-associated bacterial and fungal communities, identified differentially abundant metabolites, annotated enriched metabolic pathways, and elucidated correlations between microorganisms and metabolites. Sequencing yielded 1,862,937 valid bacterial tags and 1,757,276 valid fungal tags, which were clustered into 11,698 bacterial ASVs and 4,011 fungal ASVs. Fertilization regimes substantially altered microbial community composition, with Pseudomonadota and Ascomycota as the dominant bacterial and fungal phyla, respectively. A total of 355 known metabolites were identified, predominantly enriched in xenobiotic biodegradation and amino acid metabolism pathways. T3 exhibited the highest number of differentially abundant metabolites (206) compared to CK, with significant enrichment in pyrimidine and phenylalanine metabolism. Correlation analysis revealed significant treatment-specific associations between key rhizosphere bacterial genera and differentially abundant metabolites (e.g., pyrimidine- and phenylalanine-pathway intermediates), with positive correlations dominating in T3 and negative correlations dominating in T2. Partial least squares path modeling (PLS-PM) further showed that fertilization affected soil metabolite accumulation predominantly through soil physicochemical properties, while the microbial-community pathway to metabolite accumulation was not statistically supported. Collectively, our findings demonstrate that fertilization exerts treatment-specific regulatory effects on root-associated microbial communities and reshapes the soil metabolome, thereby providing a theoretical basis for precision fertilization and sustainable soil management in sweet cherry orchards.

## 1 Introduction

Sweet cherry (*Prunus avium* L.) is an economically important deciduous fruit tree and a core cash crop in northern China (Hirzel, 2025). By 2024, China’s sweet cherry cultivation area had reached 258,000 hectares, ranking first globally (Blan et al., 2024). The species is mainly cultivated under open-field and protected conditions. However, open-field production is highly vulnerable to combined abiotic and biotic stresses that severely restrict fruit yield and industrial sustainability (Luo et al., 2026). Protected cultivation can effectively buffer against adverse environmental fluctuations and stabilize cherry productivity through precise environmental regulation, standardized water and fertilizer management, and scientific canopy pruning. Nevertheless, it requires sophisticated cultivation techniques and exhibits a higher failure rate than open-field production (Overbeck et al., 2017a; b). In practice, insufficient professional expertise among growers frequently leads to irrational fertilization, excessive irrigation, imprecise climate regulation, and inadequate orchard management. Specifically, excessive nitrogen application reduced cherry yield by 20-50%, while imbalanced phosphorus and potassium supply caused 30-60% yield loss. Micronutrient deficiencies (e.g., boron and zinc) and inappropriate fertilization timing further led to yield declines of 10-30% and 15-40%, respectively (Wojcik and Wojcik, 2006; Yener and Altunta, 2020; Ucgun et al., 2024; Huang et al., 2018). Moreover, such irrational agricultural practices induce secondary soil salinization and soil degradation via soluble salt accumulation and disruption of soil microbial communities. These disturbances consequently result in shallow root systems, poor root-shoot balance, uneven fruit quality, and a higher incidence of fruit cracking (Tao et al., 2026; Ting et al., 2010). Therefore, developing science-based and rational fertilization strategies is critical to achieving stable and high-quality production of protected-cultivated sweet cherry.

Soil microorganisms constitute core functional components of the rhizosphere microecosystem, actively participating in critical ecological processes such as organic matter decomposition, nutrient cycling and transformation, and suppression of plant pathogens (Wu et al., 2024; Philippot et al., 2024). The structural composition and diversity of soil microbial communities serve as key indicators for assessing soil health status and evaluating ecological functional potential (Garbeva et al., 2004). Fertilization practices significantly modify soil physicochemical properties (e.g., nutrient availability, C/N ratio, and pH), thereby reshaping the structure and metabolic functions of the rhizosphere microbial community (Gong et al., 2020; Niu et al., 2022; Yuan et al., 2023; Liu et al., 2023);. This study selected three representative soil amendments, including organic fertilizers (sheep manure and chicken manure), biochar, and EM inoculant, to systematically evaluate their regulatory effects on rhizosphere microbe-metabolite networks. Organic fertilizers such as sheep and chicken manure directly supply soil organic matter and mineral nutrients, stimulating microbial proliferation and metabolite biosynthesis (Yu et al., 2025; Zhao et al., 2022; Wen et al., 2023). Biochar, a porous carbon material, improves soil water and nutrient retention and provides stable habitats for microorganisms, thereby mediating microbial community assembly and metabolic network construction (Huang et al., 2021; Agarwal et al., 2022). The EM inoculant, a commercial consortium of beneficial microorganisms (photosynthetic bacteria, lactic acid bacteria, yeasts, and actinomycetes), accelerates organic matter decomposition, suppresses soil pathogens, and secretes bioactive metabolites to directly optimize the rhizosphere microenvironment (Santos et al., 2020; Sokolova et al., 2023). Existing studies mostly test these amendments individually, while their combined regulatory effects on rhizosphere microbes in greenhouse sweet cherry remain understudied.

Rhizosphere metabolites mainly derived from root exudates regulate microbial activity via signal transduction and carbon resource supply (Zhao et al., 2022; Wen et al., 2023). Integrating microbial community analysis with metabolomics is therefore central to unraveling plant-soil feedback mechanisms. For instance, organic acids can solubilize soil phosphorus and recruit beneficial microorganisms (Huang et al., 2021); flavonoids mediate the recruitment of symbiotic microbes (Agarwal et al., 2022); and sugars/amino acids shape microbiome composition (Santos et al., 2020). Metabolite profiles respond distinctly to different agricultural management practices, as reported in blueberry orchards where organic fertilization reshaped soil microbial communities and altered amino acid metabolite accumulation (Sokolova et al., 2023). Co-analyzing microbial communities and metabolomics helps reveal plant-soil feedback mechanisms, diagnose soil degradation and design precise fertilization schemes. Current research rarely clarifies how combined application of organic manure, biochar and EM inoculant co-regulates microbe-metabolite networks in sweet cherry rhizosphere, lacking systematic comparison for targeted fertilization guidance.

To address these knowledge gaps, this study investigated how varied compound fertilization treatments shape rhizosphere microecosystems in protected sweet cherry systems. We aimed to characterize fertilization-driven shifts in rhizosphere microbial community structure and metabolic profiles, and to decipher the microbe-metabolite associations underlying these shifts. Our findings are expected to clarify the fertilization-driven regulatory mechanisms of the rhizosphere microecosystem. These insights may provide theoretical and practical support for scientific fertilization, soil conservation, and high-quality sweet cherry production.

## 2 Materials and methods

### 2.1 Experimental site and design

The soil amendment experiment was conducted at the experimental base of Gansu Academy of Agricultural Sciences located in Wenshu Village, Wenshu Town, Jiayuguan City, Gansu Province, China (98°22′E, 39°41’N; **Supplementary Figure S1**). The experiment was carried out in four solar greenhouses, each covering an area of 1000 m^2^. Sweet cherry trees were planted at a spacing of 2 m × 4 m. Four fertilization treatments were arranged in each greenhouse, with 10 trees per treatment. To ensure sample representativeness, soil samples from five adjacent trees were mixed as one composite sample, generating two composite samples per treatment within each greenhouse and eight biological replicates for each treatment in total. The soil amendments were applied between 20 and 30 December 2024 (pre-flowering stage). Soil sampling was performed in early April 2025 (pre-harvest period). During the experimental period, the greenhouse environmental conditions were strictly managed and remained relatively stable. The daytime temperature was maintained at 18-25 °C, and the nighttime temperature was kept above 5°C. The relative humidity ranged from 50% to 60%. A drip irrigation system was used to maintain soil relative water content at 60%-80% of field capacity. All trees were cultivated under natural sunlight due to the sufficient light resources in this region. Regular ventilation was conducted on sunny days to maintain the internal CO_2_ concentration at 400-800 μmol·mol^-1^.

To evaluate the combined effects of soil amendments on the rhizosphere microecosystem of greenhouse-grown sweet cherry, four treatments were established in each greenhouse: a control (CK) receiving conventional fertilization (commercial N-P-K compound fertilizer, 15-15-15, 0.8 kg·tree^-1^, applied by ditch burial), and three treatment groups (T1, T2, T3) each receiving the same conventional fertilizer plus additional amendments as detailed in **Table 1**. The organic substrates included sheep manure, chicken manure, and biochar (25% volume ratio). The EM inoculant (Effective Microorganisms; Nongfukang Biotechnology Co., Ltd., Jiangxi, China) was used as a commercial liquid concentrate. It comprised a mixed culture of photosynthetic bacteria (*Rhodopseudomonas* spp.), lactic acid bacteria (*Lactobacillus* spp.), yeasts (*Saccharomyces* spp.), and actinomycetes (*Streptomyces* spp.), at an approximate biomass ratio of 10:10:1:1. The product had a stated viable cell density of no less than 1 × 10^8^ CFU mL^-^¹. Before application, the stock solution was diluted 1:100 with clean water, and each tree was treated with 0.04 L of the diluted suspension (Zhang et al., 2013). Rhizosphere soil was collected from each treatment. High-throughput sequencing was used to characterize bacterial and fungal community diversity and differentially abundant taxa. LC-MS-based untargeted metabolomics was applied to screen differential metabolites and enriched metabolic pathways. Microbe-metabolite correlations were then explored.

**Table 1:** Fertilization materials and application rates for CK, T1, T2 and T3 (n=8).

Treatment	Fertilization & amendment composition	Application rate per tree (kg)	Application method
CK	Conventional N-P-K compound fertilizer (15-15-15)	0.8	Ditch burial
T1	Conventional fertilizer + sheep manure + biochar + EM inoculant	0.8 + 14 + 0.7 + 0.04	Ditch burial (all mixed)
T2	Conventional fertilizer + chicken manure + biochar + EM inoculant	0.8 + 14 + 0.7 + 0.04	Ditch burial (all mixed)
T3	Conventional fertilizer + biochar + EM inoculant	0.8 + 0.7 + 0.04	Ditch burial (all mixed)

### 2.2 Collection and pretreatment of rhizosphere soil samples

Rhizosphere soil was collected via the conventional root-shaking method. In December 2024, prior to the application of the amendments, a total of eight composite soil samples were collected from each of the four greenhouses, with two samples obtained per treatment. Five healthy sweet cherry trees with consistent growth status and plant size were randomly selected in each replicated plot. After clearing surface litter, root zones at the depth of 10–30 cm were carefully excavated. Fine roots (diameter < 5 mm) tightly wrapped with adherent soil were sampled and shaken vigorously to remove loose surface soil; the soil firmly attached to root surfaces was defined as rhizosphere soil. Rhizosphere soil collected from five trees in one plot was fully mixed to form a single biological replicate. Fresh soil samples were immediately placed on ice after collection and rapidly transported to the laboratory for the analysis of baseline physicochemical properties. In April 2025, rhizosphere soil samples were collected using the same method. Upon arrival at the laboratory, each sample was divided into two portions: one portion was air-dried for physicochemical analyses, and the other portion was passed through a sterile 2-mm sieve to remove coarse debris and gravel, and then stored at −80 °C for subsequent DNA extraction and high-throughput sequencing.

### 2.3 Soil physicochemical detection

Soil alkali-hydrolyzable nitrogen (AN) was determined by the alkaline diffusion procedure. Available phosphorus (AP) was extracted with sodium bicarbonate and quantified via molybdenum-antimony-ascorbic acid colorimetry. Available potassium (AK) was assayed by flame photometry, and soil organic matter (SOM) was determined by the potassium dichromate external heating oxidation method. Soil pH was determined using a pH meter in a soil-water suspension (soil: distilled water = 1:2.5, w/v) after 30 min of shaking equilibration.

### 2.4 Soil microbial community analysis

Total genomic DNA was extracted using a modified CTAB/SDS method optimized for rhizosphere soil (Zhang et al., 2013). The bacterial 16S rRNA gene was amplified using the primer pair 338F (5’-ACTCCTACGGGAGGCACCA-3’) and 806R (5’-GGACTACHVGGGTWTCTAAT-3’). For fungi, the internal transcribed spacer (ITS) region was amplified with the primer pair ITS1F (5’-CTTGGTCATTTAGAGGAAGTAA-3’) and ITS2R (5’-GCTGCGTTCTTCATCGATGC-3’) (Wang et al., 2019). PCR products were visualized via 1% agarose gel electrophoresis and purified using the GeneJET Gel Extraction Kit (Thermo Fisher Scientific). Purified amplicons were pooled in equimolar ratios to construct sequencing libraries, which were subsequently sequenced on the Illumina NovaSeq 6000 platform to generate 250-bp paired-end reads. Raw paired-end reads were first processed with fastp v0.23.4 for adapter trimming and sliding-window quality filtering (4-bp window, mean Phred Q20 cutoff; reads failing the Q20 threshold or containing more than 5 ambiguous bases were discarded). Primer sequences were removed using cutadapt v4.6. The resulting high-quality forward and reverse reads were merged into consensus contigs with VSEARCH v2.27.0 (-fastq_mergepairs, minimum merged length 200 bp, maximum mismatch rate 5%). Chimeric contigs were identified and removed with VSEARCH uchime_ref against the SILVA 138/UNITE 9.0 reference databases. Quality-filtered reads were denoised and inferred as amplicon sequence variants (ASVs) using the DADA2 pipeline (version 1.30) (Callahan et al., 2016). Taxonomic annotation of ASVs was performed using the naïve Bayesian classifier implemented in QIIME2 v2023.9 (q2-feature-classifier) (Bolyen et al., 2019), based on the SILVA 138 (16S) and UNITE 9.0 (ITS) database with a confidence threshold of 70% (Quast et al., 2013). To minimize the confounding effect of uneven sequencing depth across samples, all samples were rarefied to a uniform depth of 50,000 high-quality reads per sample before alpha- and beta-diversity analyses (random subsampling without replacement); samples below this threshold were excluded. Rarefaction curves were generated using R (v.4.3.2) by subsampling sequences in increments of 500 reads and calculating the expected ASV richness at each depth; curves approached saturation for all samples (**Supplementary Figure S2**), confirming that sequencing depth was sufficient to capture the majority of community diversity. Alpha diversity indices (Chao1, Observed ASVs, Shannon, and Simpson) were compared among treatments using the Kruskal-Wallis test implemented in R (v.4.3.2). Beta diversity was assessed by principal coordinate analysis (PCoA) based on Bray-Curtis distances computed in R (v.4.3.2) using the vegan package (v2.6-4) and visualized with the ggplot2 package (v3.4.4).

### 2.5 Nontargeted metabolomics analysis

For metabolomic analysis, 0.5 g of frozen rhizosphere soil was extracted with 1 mL of pre-chilled 80% methanol (containing 0.1% formic acid) by vigorous vortexing for 2 min. The mixture was incubated on ice for 10 min, then centrifuged at 12,000 × g for 10 min at 4 °C. The supernatant was collected and filtered through a 0.22 μm PTFE membrane filter into a glass vial before LC-MS analysis. Nontargeted metabolomics was performed on an LC-MS system consisting of a Waters Acquity I-Class Ultra Performance Liquid Chromatograph coupled to a Waters Xevo G2-XS Quadrupole Time-of-Flight High-Resolution Mass Spectrometer. Chromatographic separation was achieved on a Waters Acquity UPLC HSS T3 column (1.8 μm, 2.1 × 100 mm). The mobile phase conditions were as follows: for both positive and negative ion modes, mobile phase A was 0.1% formic acid in water, and mobile phase B was 0.1% formic acid in acetonitrile. The injection volume was 1 μL (Dunn et al., 2011). Primary and secondary mass spectrometry data were acquired in MSe mode using the Waters Xevo G2-XS QToF mass spectrometer controlled by MassLynx V4.2 software (Li et al., 2024b). During each acquisition cycle, dual-channel data collection was performed simultaneously at low and high collision energies. Low collision energy was set at 2 eV, and high collision energy ranged from 10 to 40 eV, with a scan time of 0.2 s per spectrum. Electrospray ionization (ESI) source parameters were configured as follows: capillary voltage, 2000 V (positive mode) or -1500 V (negative mode); cone voltage, 30 V; ion source temperature, 150 °C; desolvation gas temperature, 500 °C; purge gas flow rate, 50 L·h^-1^; desolvation gas flow rate, 800·h^-1^. Raw data collected by MassLynx V4.2 were processed using Progenesis QI software (version 2.0) for peak detection, alignment, and normalization. Metabolite identification was performed using two standard mass spectral databases: the BGI High Resolution Accurate Mass Plant Metabolome Database (BGI HRAM-PMDB), an in-house database established by BGI (Shenzhen, China), and the mzCloud database (https://www.mzcloud.org). Identification was based on theoretical fragment matching and mass error tolerance within 100 ppm.

### 2.6 Data analysis

Prior to statistical comparison, data were checked for normality and homogeneity of variance using Shapiro-Wilk and Bartlett’s tests, respectively. All five soil physicochemical indicators (pH, SOM, AN, AP, AK) conformed to normal distribution (Shapiro-Wilk P > 0.05) and variance homogeneity (Bartlett P > 0.05) across treatments after log_10_ transformation where required. To partition treatment-induced net changes from initial soil heterogeneity, the greenhouse-level baseline value of each indicator (**Supplementary Table S1**) was included as a covariate in a linear mixed-effects model implemented in lme4: lmer as post ~ treatment + baseline_value + (1 | greenhouse) (Bates et al., 2015), with P-values obtained via lmerTest:: anova () and Kenward-Roger degrees-of-freedom correction (Kuznetsova et al., 2017). Greenhouse was modeled as a random effect to absorb block-level variation, and Tukey HSD *post-hoc* contrasts were used for pairwise treatment comparisons. For alpha diversity indices (Chao1, Observed, Shannon, Simpson) and PCoA-derived beta-diversity distances, the same LMM framework was applied without baseline covariates because microbial composition was not measured at pre-treatment.

PCoA based on Bray-Curtis distances was performed to visualize differences in microbial community structure among treatments, with statistical significance assessed by permutational multivariate analysis of variance (PERMANOVA, vegan:: adonis2) and analysis of similarities (ANOSIM, vegan:: anosim) (Oksanen et al., 2022). Given that PERMANOVA and ANOSIM are susceptible to both location and dispersion effects (Anderson, 2001; Anderson et al., 2010; Warton et al., 2012), we further tested the homogeneity of group dispersions using the betadisper function in the vegan package with type = “centroid”, and assessed its significance via a permutation test (9,999 permutations). This procedure enabled us to distinguish whether the observed significant differences were attributable to genuine centroid shifts or to heterogeneity in within-group variability. Differential abundance analysis was conducted using the Linear Discriminant Analysis Effect Size (LEfSe) method implemented in the microbiomeMarker R package (Segata et al., 2011; Cao et al., 2022), with a Kruskal-Wallis alpha of 0.05 among classes and a logarithmic LDA score threshold of 3.5. No additional multiple-testing correction was applied for LEfSe, because the method internally controls false discoveries through a strict sequential-testing strategy. The RCbray (Raup-Crick with Bray-Curtis dissimilarity) was used to characterize stochastic assembly processes, following Stegen et al. (2013). Pairwise RCbray values were calculated using the RaupCrick implementation (1000 randomizations) in the R package micEco, with values constrained to [−1, +1]. Following Chase et al. (2011) and Stegen et al. (2013), pairwise comparisons were partitioned into three stochastic categories: dispersal limitation (RCbray > 0.95), homogenizing dispersal (RCbray < −0.95), and undominated processes (|RCbray| ≤ 0.95; encompassing ecological drift, weak selection and unmeasured deterministic forces). Co-occurrence networks were constructed using the dominant phylotypes recovered from all samples. For each of the four treatments, bacterial and fungal taxa with mean relative abundances greater than 0.05% were retained for network analysis. Pairwise correlations were calculated using Spearman’s rank coefficient, and only those with correlation coefficients (r) above 0.65 and FDR-corrected P-values below 0.001 were considered statistically robust and retained for network construction (Barberán et al., 2012). Network topological parameters were computed with the “igraph” package, and all networks were visualized in Cytoscape v3.7.1 (Shannon et al., 2003; https://cytoscape.org). To assess the non-randomness of network structure, each observed network was compared with 100 randomized networks preserving the same number of nodes and edges, generated using the Network Randomizer tool within Cytoscape. Genera with the highest betweenness centrality were designated as keystone taxa, following the criterion that high betweenness reflects a node’s pivotal role in mediating connections within the network (Vick-Majors et al., 2014). Network complexity was quantified as the averaged Z-score derived from multiple normalized topological metrics. Identified metabolites were annotated and mapped to metabolic pathways using the HMDB (Wishart et al., 2018), KEGG (Kyoto Encyclopedia of Genes and Genomes) (Ogata et al., 1999), and LipidMaps databases (Fahy et al., 2007). Orthogonal Partial Least Squares Discriminant Analysis (OPLS-DA) was performed using SIMCA-P (version 14.1, Umetrics, Sweden) and MetaX for metabolomics data. The resulting model performance metrics were R²Y > 0.9 and Q² > 0.5. Variable Importance in Projection (VIP) values were calculated via multiple cross-validations. Differential metabolites were selected based on a combination of fold change (> 1.5 or < 0.67), *P*-value (< 0.05), and VIP value (> 1.0). The *P*-value for each metabolite was calculated using a two-tailed Student’s t-test (for normally distributed data) or Wilcoxon rank-sum test (for non-normally distributed data), after checking normality with the Shapiro-Wilk test. To control the false discovery rate (FDR), the Benjamini-Hochberg procedure was applied, and metabolites with FDR-adjusted P-value < 0.05 were considered statistically significant.

Spearman’s correlation analysis was performed using genus-level relative abundance data of microorganisms and normalized metabolite features from the metabolome. The Benjamini-Hochberg false discovery rate (BH-FDR) method was applied to correct for multiple comparisons, and correlations with |r| ≥ 0.7 and FDR < 0.05 were selected as the display threshold. To explore the statistical associations among fertilization, soil physicochemical properties, microbial communities and the metabolome, partial least squares path modeling (PLS-PM) was applied as an exploratory path analysis. PLS-PM was chosen because it tolerates the small sample size (n = 32) and does not require multivariate normality. Four latent blocks were specified: fertilization (three treatment levels were coded relative to CK), soil physicochemical properties (five indicators), microbial communities (modeled separately using five dominant bacterial and fungal genera), and soil metabolites (16 KEGG-annotated differential metabolites). Separate bacterial and fungal models was fitted to avoid collinearity. Given the small sample size (n = 32) and the indicator-to-sample ratio of the metabolite block, the path estimates should be interpreted with caution. Models were fitted with reflective measurement specifications using the R package plspm (v 0.5.1), with all manifest variables z-score standardized prior to analysis to enable direct comparisons of path coefficients. Path coefficients were evaluated for significance using 500 bootstrap resamples (nonparametric percentile method), and p-values were derived from a two-sided z-test of the bootstrapped path coefficients. Model quality was assessed using the composite reliability (CR), average variance extracted (AVE) for convergent validity, the heterotrait-monotrait (HTMT) ratio for discriminant validity (threshold HTMT < 0.85, following Franke and Sarstedt, 2019), the coefficient of determination (R²) for endogenous constructs, and the goodness-of-fit (GoF) index for the overall model (**Supplementary Table S2**).

## 3 Results

### 3.1 Soil physicochemical characteristics of greenhouse sweet cherry rhizosphere soil

Soil pH, SOM, and AN, AP, and AK were measured before and after treatment application (**Figure 1**; **Table S1**). Baseline homogeneity across the four greenhouses was confirmed for all indicators (one-way ANOVA, all P > 0.05; CV: 0.24 – 3.12%), with starting values being nearly identical among blocks (e.g., AN = 55.40 ± 1.64 mg·kg^-^¹; AK = 133.25 ± 3.23 mg·kg^-^¹). Linear mixed-effects models with baseline as a covariate and greenhouse as a random intercept were therefore used to isolate treatment effects from initial spatial variation. The greenhouse random effect accounted for 8 – 14% of total variance (conditional R²), indicating effective control of block heterogeneity.

**Figure 1:**
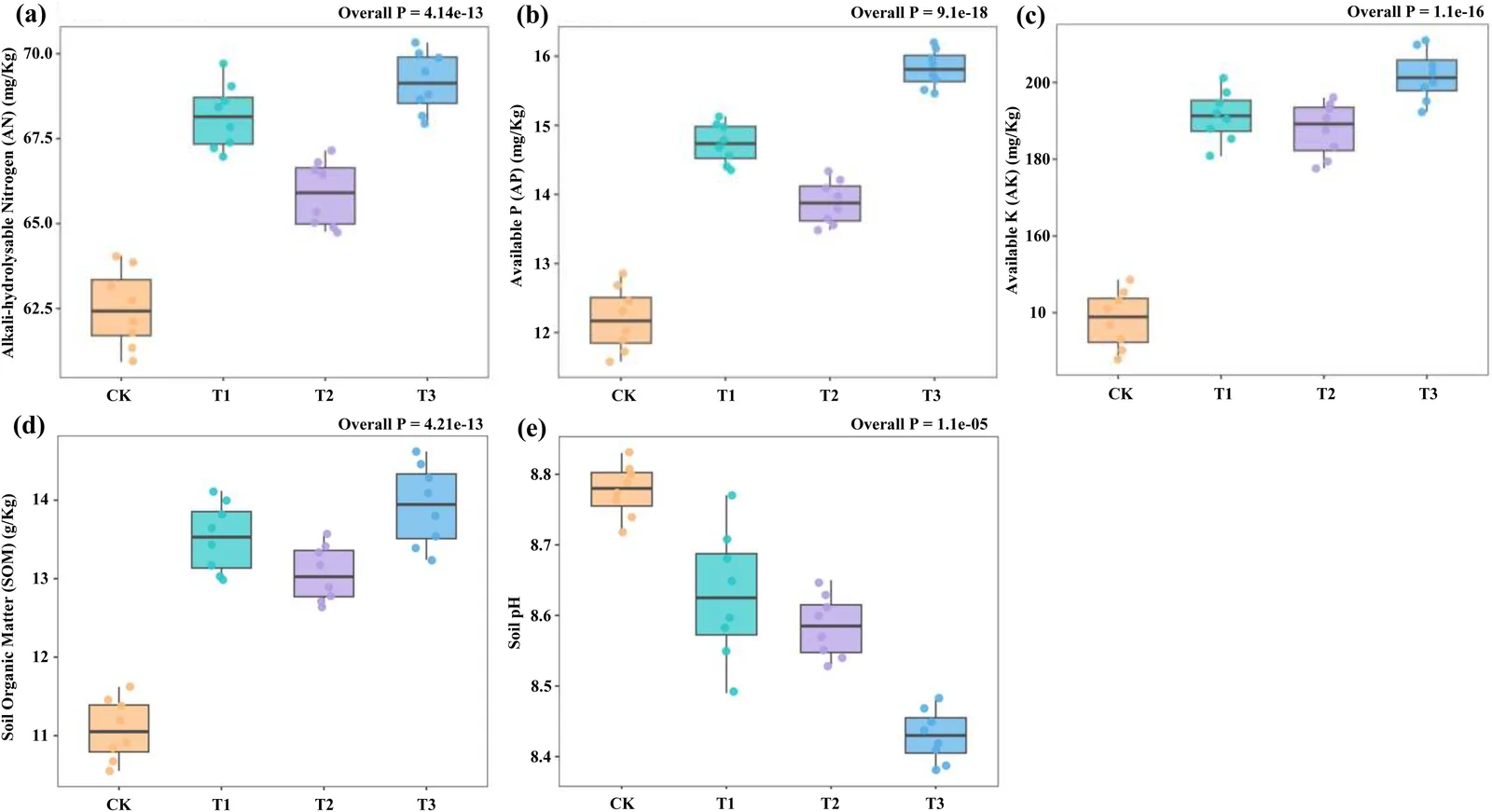
Changes in key soil physicochemical properties under different fertilization treatments of greenhouse sweet cherry. Boxplots illustrate the variations of **(A)** alkali-hydrolysable nitrogen (AN), **(B)** available phosphorus (AP), **(C)** available potassium (AK), **(D)** soil organic matter (SOM) and **(E)** soil pH across CK, T1, T2 and T3 groups. Overall P values derived from one-way ANOVA (AN, AP, AK, SOM) or Kruskal-Wallis test (pH) are marked on each subgraph to indicate significant inter-treatment differences.

After fertilization treatment, median concentrations of AN increased relative to CK by 9.1%, 5.6%, and 10.9% in T1, T2, and T3, respectively. For AP, the corresponding increases were 20.9%, 13.9%, and 29.5%. Median AK rose by 6.7%, 5.6%, and 12.3%, respectively. Kruskal–Wallis tests confirmed that treatment effects were highly significant for all three nutrients (**Figures 1A–C**). SOM responded to the amendments with a modest but statistically detectable increase of 6–11% (*P* < 0.05) (**Figure 1D**), whereas pH showed a slight decline, decreasing by 0.12 to 0.18 across the three treatments (*P* < 0.05) (**Figure 1E**). Pairwise comparisons (Tukey HSD) further revealed that T3 (biochar + EM) produced the highest AN and AP levels, significantly exceeding those in T1 and T2 (*P* < 0.05), despite receiving no animal manure.

### 3.2 Sequencing data and characteristics of microbial community richness and diversity

High-throughput sequencing of the 16S rRNA and ITS gene regions was conducted to characterize rhizosphere soil microbial communities in sweet cherry under different fertilization regimes. Following rigorous quality control, including the removal of low-quality sequences and chimeric reads, a total of 1,862,937 valid bacterial tags (mean: 58,216 clean tags per sample; range: 54,962-61,552) and 1,757,276 valid fungal tags (mean: 54,914 clean tags per sample; range: 43,091-64,352) were obtained. ASVs were clustered using DADA2 pipeline at single-nucleotide resolution, resulting in 11,698 bacterial ASVs and 4,011 fungal ASVs overall. A total of 3,876 bacterial ASVs and 152 fungal ASVs shared across all four treatment groups (**Figures 2A, B**). These findings indicate partial overlap in the core microbial taxa across treatments, while also highlighting distinct compositional differentiation among microbial communities under varying fertilization practices.

**Figure 2:**
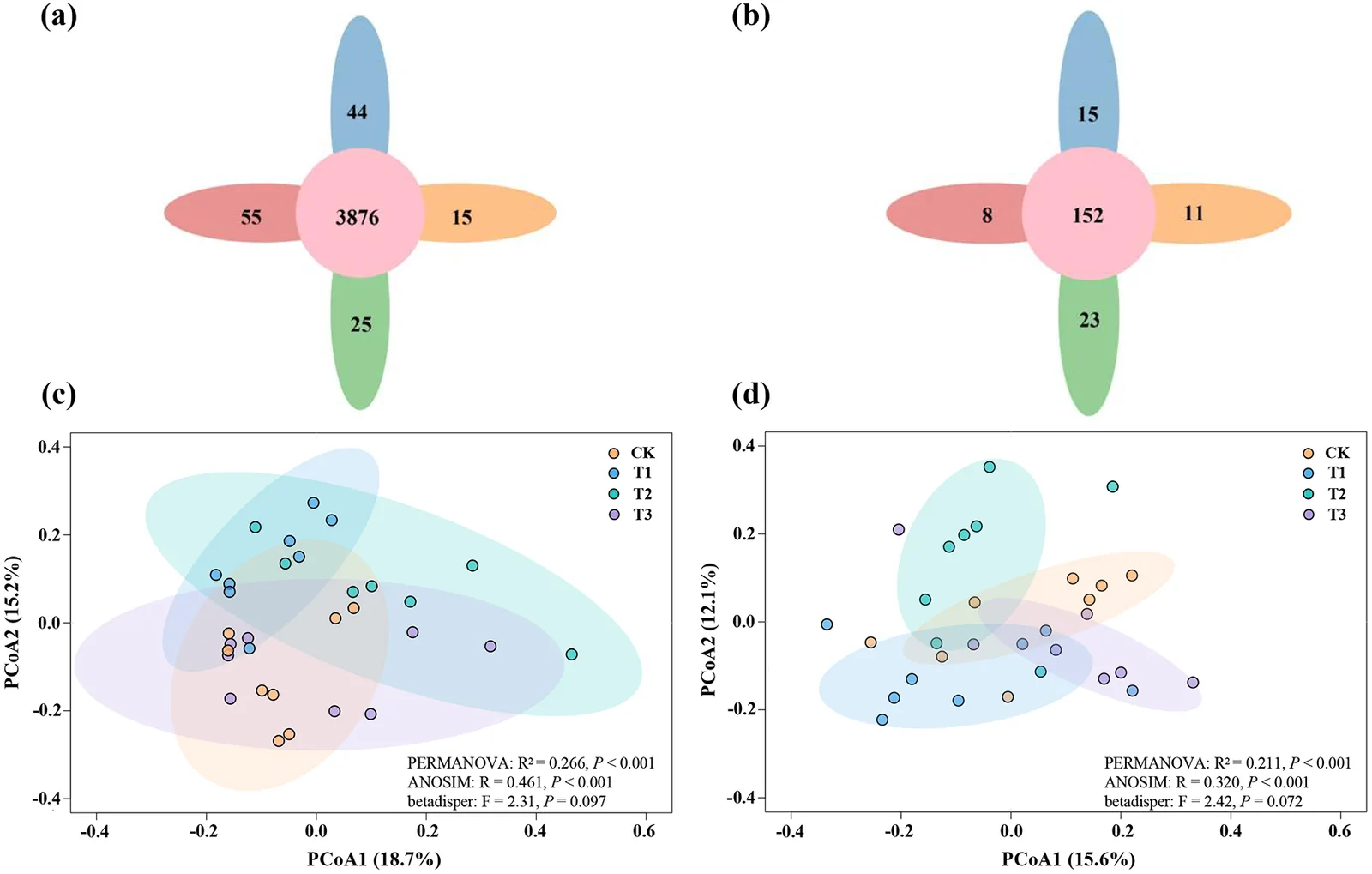
Sequencing data of root-associated microorganisms in sweet cherry under different fertilization treatments. Petal diagrams of ASV distribution for soil bacteria **(A)** and fungi **(B)** at different sampling sites. Principal coordinate analysis (PCoA) based on Bray–Curtis dissimilarity showing the community composition of bacteria **(C)** and fungi **(D)** under different fertilization treatments. Ellipses represent 95% confidence intervals around group centroids. Both PERMANOVA and ANOSIM revealed significant separation among treatments (P < 0.001 for both bacterial and fungal communities), while betadisper tests confirmed no significant differences in within-group dispersion, indicating that the observed compositional shifts reflect genuine changes in community centroids rather than heterogeneity of variances.

Alpha diversity indices were calculated at the ASV level to assess microbial community richness and diversity (**Supplementary Figure S2**; **Supplementary Table S3**). There were no significant differences in the Chao1, Observed, Shannon, and Simpson indices of soil bacterial and fungal communities among different treatments (CK, T1, T2, T3), indicating that each treatment had no significant effect on the species richness and diversity of soil microbial communities. Additionally, rarefaction curves for both bacterial and fungal communities approached saturation (**Supplementary Figure S3**), indicating sufficient sequencing depth to capture the majority of microbial diversity and supporting the reliability of downstream analyses.

To further investigate the impact of fertilization on microbial community structure, principal coordinate analysis (PCoA) based on Bray-Curtis distances was performed at the ASV level (**Figures 2C, D**). For bacterial communities, PCoA1 and PCoA2 explained 15.2% and 18.7% of the total variation, respectively; for fungal communities, PCoA1 and PCoA2 explained 15.6% and 12.1%, respectively. PERMANOVA revealed that treatment effects accounted for a significant proportion of the variation in both bacterial (R² = 0.266, *P* < 0.001) and fungal (R² = 0.211, *P* < 0.001) communities, and ANOSIM further confirmed significant rank-based dissimilarities among groups (bacterial R = 0.461, *P* < 0.001; fungal R = 0.320, *P* < 0.001). However, ordination plots showed considerable overlap in the 95% confidence ellipses across the four treatments, with only a modest displacement of the CK centroid from the amended groups along PCoA1, suggesting that the centroid separation, while statistically meaningful, was limited in magnitude. Betadisper tests indicated no significant differences in within-group dispersion among treatments for either bacteria (F = 2.31, *P* = 0.097) or fungi (F = 2.42, *P* = 0.072; permutest with 9999 permutations), confirming that the significant PERMANOVA and ANOSIM results were attributable to genuine shifts in community centroids rather than to heterogeneous multivariate spread. Nevertheless, the relatively low effect sizes and the substantial overlap of ellipses suggest that, although fertilization induced statistically significant alterations in microbial community composition, the segregation among treatments remained constrained in the reduced ordination space. Collectively, the combined application of organic amendments and EM inoculant significantly modified the overall microbial community structure relative to the control, albeit with only moderate between-group differentiation.

### 3.3 Community composition analysis based on 16S rRNA genes (bacteria) and ITS genes (fungi)

Analysis of soil microbial community composition under different fertilization treatments revealed that bacterial sequences were classified into 36 phyla, 80 families, and 95 genera, while fungal sequences were assigned to 12 phyla, 46 families, and 79 genera. Notably, different fertilization regimes altered the relative abundance of rhizosphere bacterial communities.

### 3.4 Bacterial communities

The phylum-level distribution of soil bacteria differed among fertilization treatments. A total of 19 bacterial phyla were detected across all samples. Among them, 12 core phyla occupied 97.31%-98.25% of the total sequences (**Figure 3A**). Pseudomonadota was the most abundant phylum in all treatments, with relatively higher proportions in T2 and CK, and lower levels in T1 and T3. Acidobacteriota showed an opposite trend, presenting lower relative abundance in T2 and CK, but higher values in T1 and T3. Pseudomonadota emerged as the dominant phylum across all treatments, while Acidobacteriota was consistently present as a subdominant taxon in all samples (**Supplementary Figure S4A**), a pattern consistent with the typical distribution of major bacterial taxa in orchard soils. The genus-level composition of soil bacteria differed among fertilization treatments. Dominant bacterial genera were identified across all samples (**Figure 3B**). Among the prevailing genera, Gp6 (19.95%-28.51%) was consistently abundant in all treatments, with slightly higher proportions in T2 and T3. Fertilization treatments influenced the relative abundances of *Stenotrophobacter* and *Sphingomonas* in T2 and T3. Furthermore, LEfSe analysis (LDA score ≥ 3.5, *P* < 0.05) identified 65 bacterial biomarkers across multiple taxonomic levels (phylum to species) (**Supplementary Figure S4B**). The T2 treatment had the most enriched taxa, with Actinomycetota showing the highest LDA score. The T1 treatment was mainly enriched in Flavobacteriia and Vibrionales-related groups, while the T3 treatment was enriched in Caulobacterales and Deltaproteobacteria. Only a few taxa were enriched in the CK group, indicating that each treatment induced specific bacterial biomarkers.

**Figure 3:**
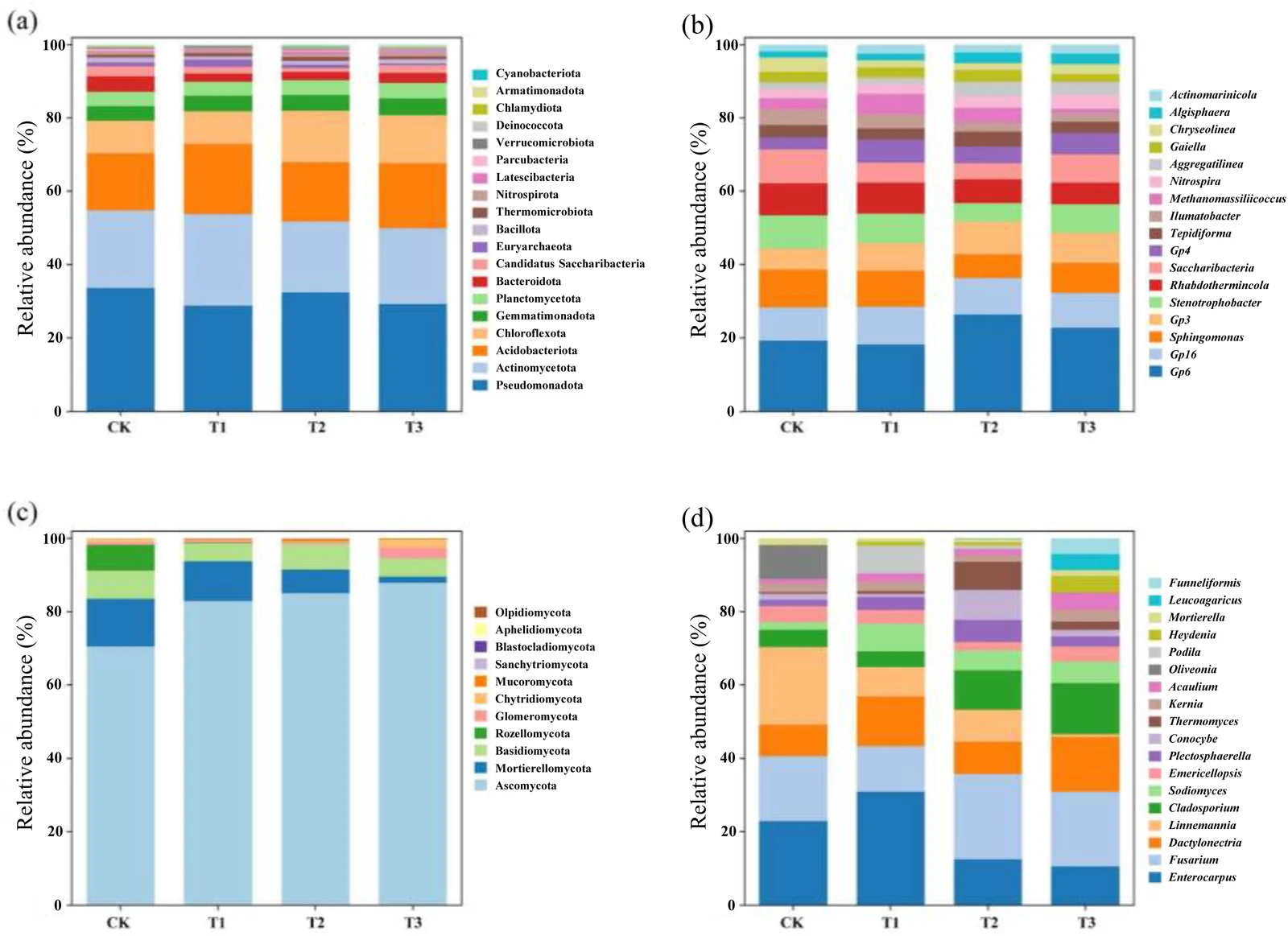
Analysis of microbial community composition among different treatments. A bar graph displays the relative abundance of rhizosphere bacteria **(A)** and fungi **(C)** in sweet cherry under different conditions at the phylum level. A bar graph displays the relative abundance of rhizosphere bacteria **(B)** and fungi **(D)** in sweet cherry under different conditions at the genus level.

### 3.5 Fungal communities

Fungal relative abundance also varied across soil samples of the control and fertilization treatments. A total of 11 fungal phyla were identified in all samples. Seven dominant phyla accounted for 99.03%-99.97% of total sequences. Fungal sequences that could not be classified at the phylum level were grouped as unclassified fungi. *Ascomycota* was the most abundant phylum across all groups, showing higher proportions in T2 (87.15%) and T3 (90.02%). Fertilization generally increased the relative abundance of Ascomycota. Basidiomycota exhibited intermediate abundance among treatments, while Mortierellomycota was more abundant in the CK than in fertilized treatments (**Figure 3C**). These changes may be attributed to fertilization-induced alterations in soil carbon-to-nitrogen ratio and improved nutrient availability. Basidiomycota persisted as a subdominant phylum across all treatment groups (**Supplementary Figure S5A**). At the genus level, dominant fungi included *Enterocarpus*, *Fusarium, Dactylonectria*, *Linnemannia*, and *Cladosporium* (**Figure 3D**). The T2 and T3 treatments had increased relative abundances of *Fusarium*, *Cladosporium*, and *Dactylonectria*, accompanied by reduced relative abundances of *Enterocarpus* and *Linnemannia*. This LEfSe analysis plot displays fungal biomarkers with significant differences across treatments (LDA ≥ 3.5, P < 0.05, **Supplementary Figure S5B**). The CK had the most enriched taxa, dominated by Mortierellomycota-related groups. The T1 was mainly enriched in *Microascales* and *Microascaceae*. The T2 showed enrichment of *Thermomyces* and *Mucor*. Only two taxa (*Pseudombrophilaceae* and *Funneliformis*) were enriched in the T3 treatment. Specific fungal biomarkers were identified for each treatment.

### 3.6 Microbial co-occurrence complexity and community assembly processes

To uncover how different fertilization practices reshaped microbial interspecific associations and community assembly, genus-level co-occurrence network topological indices of bacteria and fungi were quantified, including clustering coefficient, network density, total edge number, average degree and modularity (**Figure 4**). For bacterial communities, CK exhibited the highest clustering coefficient, edge number, network density and average degree among all groups, whereas these four complexity-related topological metrics gradually declined from CK to T3. By contrast, modularity presented a rising trend along the gradient of CK, T1, T2 and T3, implying fertilization reduced overall bacterial network complexity while strengthening the modular partitioning of bacterial community (**Figure 4A**). In fungal co-occurrence networks, T2 possessed the maximum values of clustering coefficient, edge number, density and average degree, which were distinctly higher than CK, T1 and T3, demonstrating T2 fertilization may elevate fungal network complexity. Meanwhile, fungal modularity gradually decreased from CK to T3, indicating fertilization weakened modular structure of fungal community (**Figure 4B**). To facilitate intuitive assessment of topological metrics and to verify the identification of edges, modules, and hub nodes, **Supplementary Figure S6** visualizes the actual bacterial and fungal co-occurrence networks under each treatment. The visualized networks consistently demonstrate that, across all treatments, bacterial networks possess a greater number of nodes and edges, as well as higher overall density, compared to fungal networks.The result indicated that the RCbray values of fungi were consistently positive, while bacterial RCbray values were the opposite in all treatments (**Figure 4C**). Notably, nearly all pairwise comparisons for both groups exhibited |RCbray| < 0.95, indicating that stochastic community turnover was primarily governed by undominated ecological drift, with smaller fractions attributed to dispersal limitation and minimal homogenizing dispersal across all treatments (**Figure 4D**). Fungal communities exhibited a higher dispersal-limitation fraction than bacterial communities, suggesting stronger stochastic turnover in fungi, likely reflecting fungal dispersal limitation and weaker environmental filtering in this perennial-crop rhizosphere. Overall, undominated stochastic processes (drift and weak selection) dominated microbial community turnover across treatments, while a secondary dispersal-limitation signal was detected, especially in fungal communities. Bacteria exhibited a relatively higher undominated fraction than fungi, indicating that bacterial communities were more influenced by weak/unmeasured deterministic forces, consistent with their denser co-occurrence networks and stronger environmental filtering.

**Figure 4:**
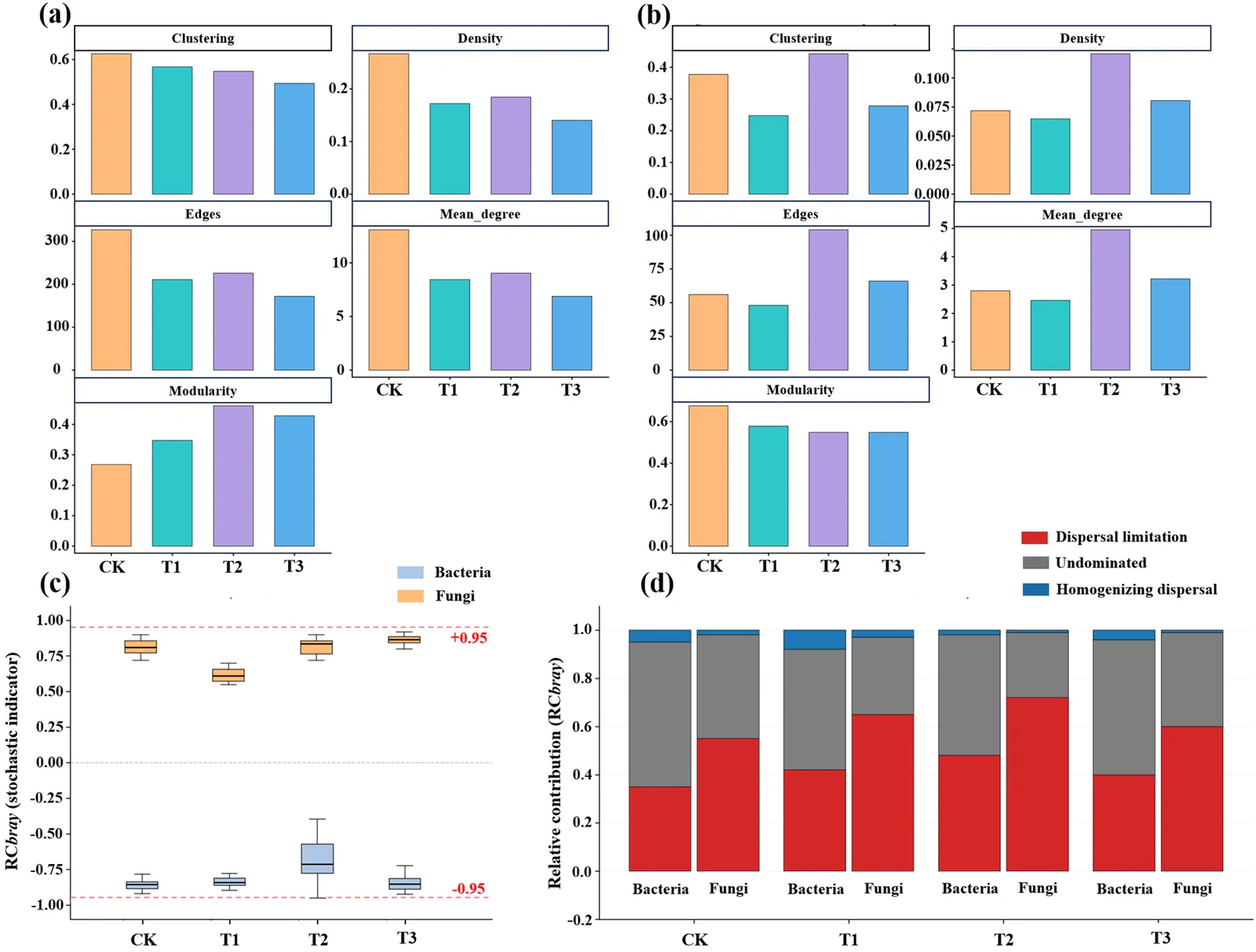
Co-occurrence network complexity and stochastic community assembly patterns of rhizosphere bacterial and fungal communities. **(A)** Topological parameters of bacterial co-occurrence networks, including clustering coefficient, network density, edge number, average degree and modularity across CK, T1, T2 and T3 treatments. **(B)** Topological parameters of fungal co-occurrence networks. **(C)** RC_bray_ values derived from the Bray-Curtis null model, characterizing the stochastic component of community assembly for bacteria and fungi. **(D)** Relative contributions of dispersal limitation (DL), undominated processes (UD), and homogenizing dispersal (HD) to bacterial and fungal community assembly, expressed as the proportions of stochastic processes estimated from the Bray–Curtis null model.

### 3.7 Screening of differential rhizosphere microorganisms under different fertilization treatments

Nonmetric Multidimensional Scaling (NMDS) ordinations indicated partial separation of both bacterial (Stress = 0.175) and fungal (Stress = 0.238) communities among fertilization treatments (**Supplementary Figures S7A, B**; *P* < 0.05). In line with the PCoA results, the NMDS plots revealed extensive overlap among groups. The betadisper test confirmed that this pattern was not attributable to heterogeneity in within-group dispersion, suggesting that the significant test statistics primarily reflect a modest compositional shift rather than variation in multivariate spread. To evaluate treatment-specific shifts in microbial composition, the different abundances taxa were selected for comparative analysis of their average relative abundances across treatment groups. For bacteria, the 35 most abundant genera shifted in a treatment-dependent manner (**Figure 5A**). *Sphingomonas*, *Thermanaerothrix*, *Pseudarthrobacter*, *Truepera*, *Longimicrobium*, *Hyphomicrobium*, *Thauera*, *Aquicella*, *Dactylosporangium*, *Gemmatimonas*, *Ignavibacterium*, *Sulfuricaulis*, *Virgisporangium*, *Rubrivivax*, *Turicibacter*, *Sporichthya*, *Actinocorallia*, *Hypericibacter*, *Sporofaciens*, *Terrimonas*, *Kineosporia*, *Lacibacter*, *Aridibacter*, *Kineothrix*, *Oxalophagus* and Aeromonas were enriched in T1 relative to CK and T3. The opposite pattern was observed for *Rhodoplanes*, *Methylocystis*, *Microvenator*, *Thiohalospira*, *Aromatoleum*, *Pontibacter*, *Dongia*, *Cellulosimicrobium*, *Fimbriimonas* and *Roseomonas*, which were more abundant in T3 (and partly in CK) but depleted in T1 and T2. For fungi, the 12 dominant genera more clearly distinguished CK from the amended treatments (**Figure 5B**). *Linnemannia*, *Oliveonia*, *Solicoccozyma* and *Enterocarpus* were strongly enriched in CK but depleted in T3. *Thermomyces*, *Funneliformis*, *Scopulariopsis*, *Didymella* and *Enterocarpus* varied across T1–T3; *Coprinopsis* was enriched only in T1, whereas *Serendipita* remained relatively uniform, with a slight increase in T2. Overall, long-term fertilization under protected cultivation substantially restructured the soil microbiome. Bacterial responses were treatment-specific and broadly aligned with copiotroph–oligotroph differentiation, while fungal communities were more consistently suppressed across all fertilized treatments.

**Figure 5:**
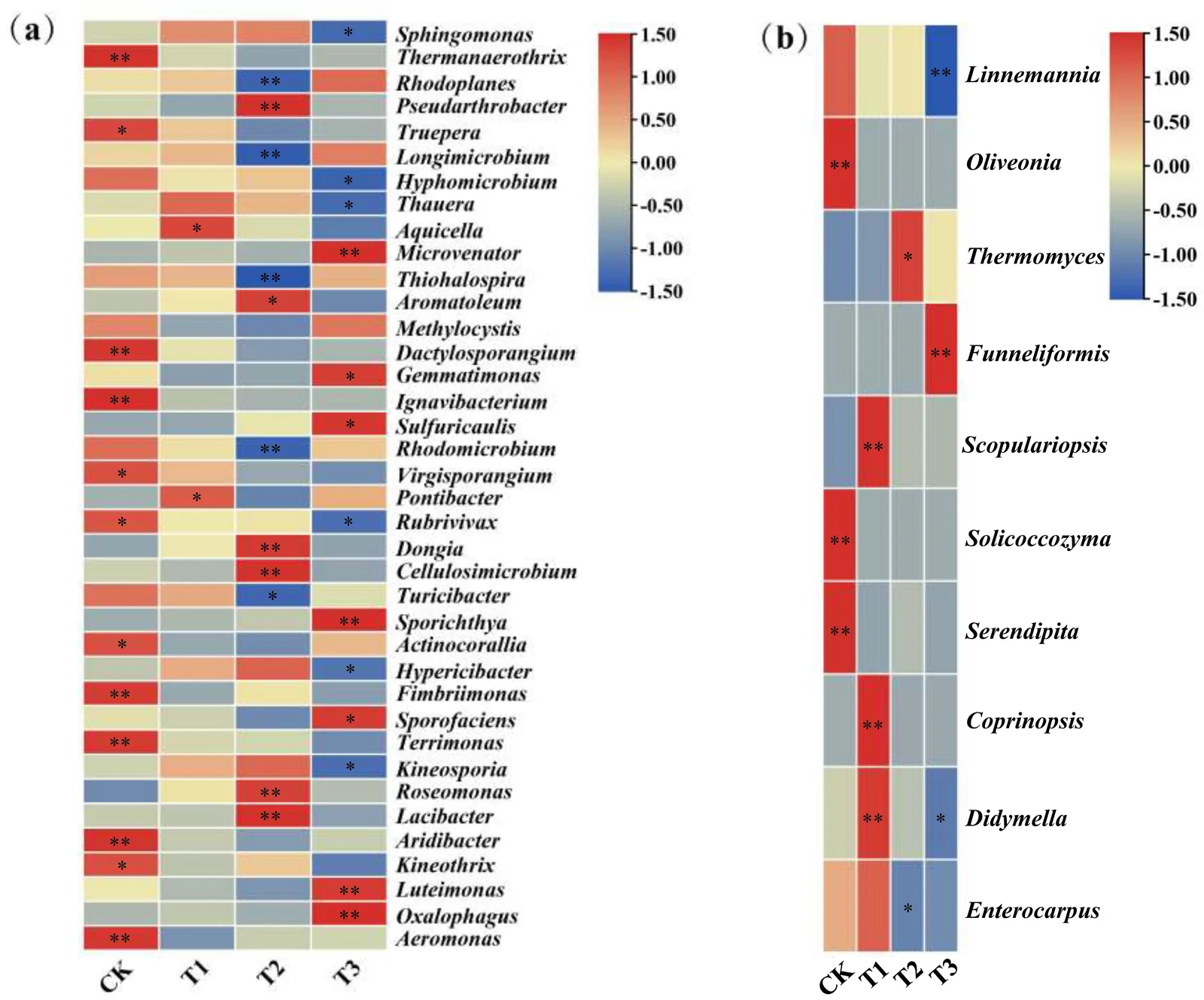
Heatmap of differentially abundant bacterial **(A)** and fungal **(B)** genera across fertilization treatments. Relative abundances of each genus were Z-score standardized across all samples to facilitate cross-treatment comparisons. Red and blue colors indicate relative enrichment and depletion, respectively, relative to the mean abundance of each genus across treatments. Statistical significance is indicated by asterisks: *P < 0.05 and **P < 0.01.

### 3.8 Characteristics of rhizosphere metabolite composition under different fertilization treatments

To investigate the response patterns of soil metabolic profiles to different fertilization regimes, untargeted metabolomics analysis was performed on 32 soil samples using an LC-MS platform. Quality control (QC) assessment of the metabolomics data was conducted by calculating the coefficient of variation (CV). Among the 14,491 detected features, 13,760 (95%) exhibited CV values ≤ 30%, indicating good repeatability of the dataset (**Supplementary Table S4**). Consistently, QC samples clustered tightly in the PCA ordination plot (**Figure 6A**), confirming high analytical stability and reliable data quality. Principal component analysis (PCA) further revealed that the first two principal components (PC1 and PC2) collectively explained 39.5% of the total variation in metabolites, sufficiently capturing the metabolic differences among treatment groups.

**Figure 6:**
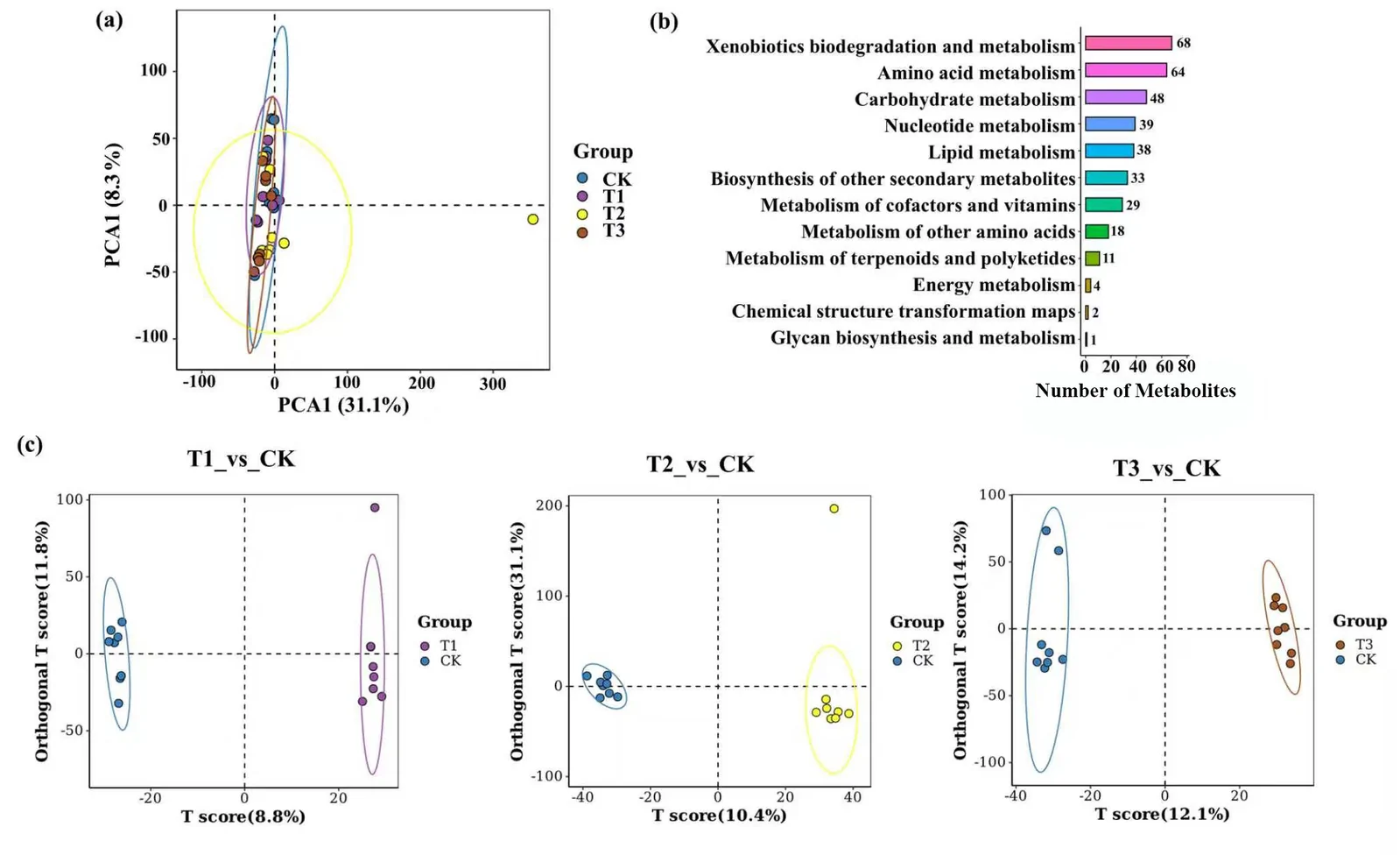
Characteristics of rhizosphere metabolite composition under different fertilization treatments. **(A)** PCA was performed on the quantitative values of all samples including quality control (QC) samples in the total data matrix, to observe the overall distribution of samples in each group and QC samples. **(B)** For the identified metabolites, the number of metabolites was counted according to the major categories of KEGG metabolic pathways they involved in. In the plot, the ordinate represents the categories of metabolic pathways, while the abscissa represents the number of metabolites. **(C)** A OPLS-DA model was established between the two sample groups to observe the distribution and separation trend of the two groups. The horizontal axis T score represents the predictive component score of the first principal component, while the vertical axis Orthogonal T score represents the orthogonal component score. Each point in the OPLS-DA score plot represents one sample, with different groups labeled in distinct colors, and the ellipses represent the 95% confidence intervals.

Metabolite identification was performed following the Metabolite Standards Initiative (MSI) criteria. Only metabolites annotated at MSI Level 1 with high confidence were used for subsequent differential analysis. The complete quantitative dataset of all detected metabolites is listed in **Supplementary Table S5**. A total of 13,760 metabolic features were detected in the dataset, among which 2,779 features were successfully annotated. All annotated metabolites were assigned to corresponding KEGG pathways and further classified into super pathways for statistical analysis. Ultimately, 355 metabolites were incorporated into this classification system. These metabolites were enriched in 13 major metabolic pathways, ranked in descending order of relative abundance as follows: xenobiotics biodegradation and metabolism (19.2%), amino acid metabolism (18.0%), carbohydrate metabolism (13.5%), nucleotide metabolism (10.9%), lipid metabolism (10.7%), biosynthesis of other secondary metabolites (9.3%), cofactor and vitamin metabolism (8.2%), metabolism of other amino acids (5.1%), terpenoid and polyketide metabolism (3.1%), energy metabolism (1.1%), chemical structure transformation maps (0.6%), and glycan biosynthesis and metabolism (0.3%) (**Figure 6B**). These pathways encompass core processes involved in soil nutrient cycling and energy dynamics. To further investigate the regulatory effects of fertilization treatments on metabolic profiles, an OPLS-DA model was constructed to compare metabolite compositions between the control group and treatment groups. OPLS-DA score plots showed visual separation between the control and fertilization treatments, as indicated by non-overlapping 95% confidence ellipses. Among the treatments, T1 and T3 displayed the most pronounced metabolic divergence relative to the control (**Figure 6C**, and **S8**). This indicates that these two fertilization regimens exerted the most pronounced influence on soil metabolite composition, providing a basis for subsequent screening of differential metabolites.

### 3.9 Identification and pathway analysis of differential rhizosphere metabolites under different fertilization treatments

The results of differential metabolite screening revealed substantial variation in the number of significantly altered metabolites between each fertilization treatments and the control (**Figure 7A**). Specifically, a total of 49 differential metabolites (14 up-regulated and 35 down-regulated) were identified in the T1_vs_CK comparison. In contrast, 23 differentially abundant metabolites were detected in T2_vs_CK, comprising 3 upregulated and 20 downregulated metabolites. The T3 treatment exhibited the highest number of differential metabolites compared to the control, with 206 biomarkers identified, 93 upregulated and 113 downregulated, indicating that the T3 regimen induced the most pronounced metabolic perturbation in the soil.

**Figure 7:**
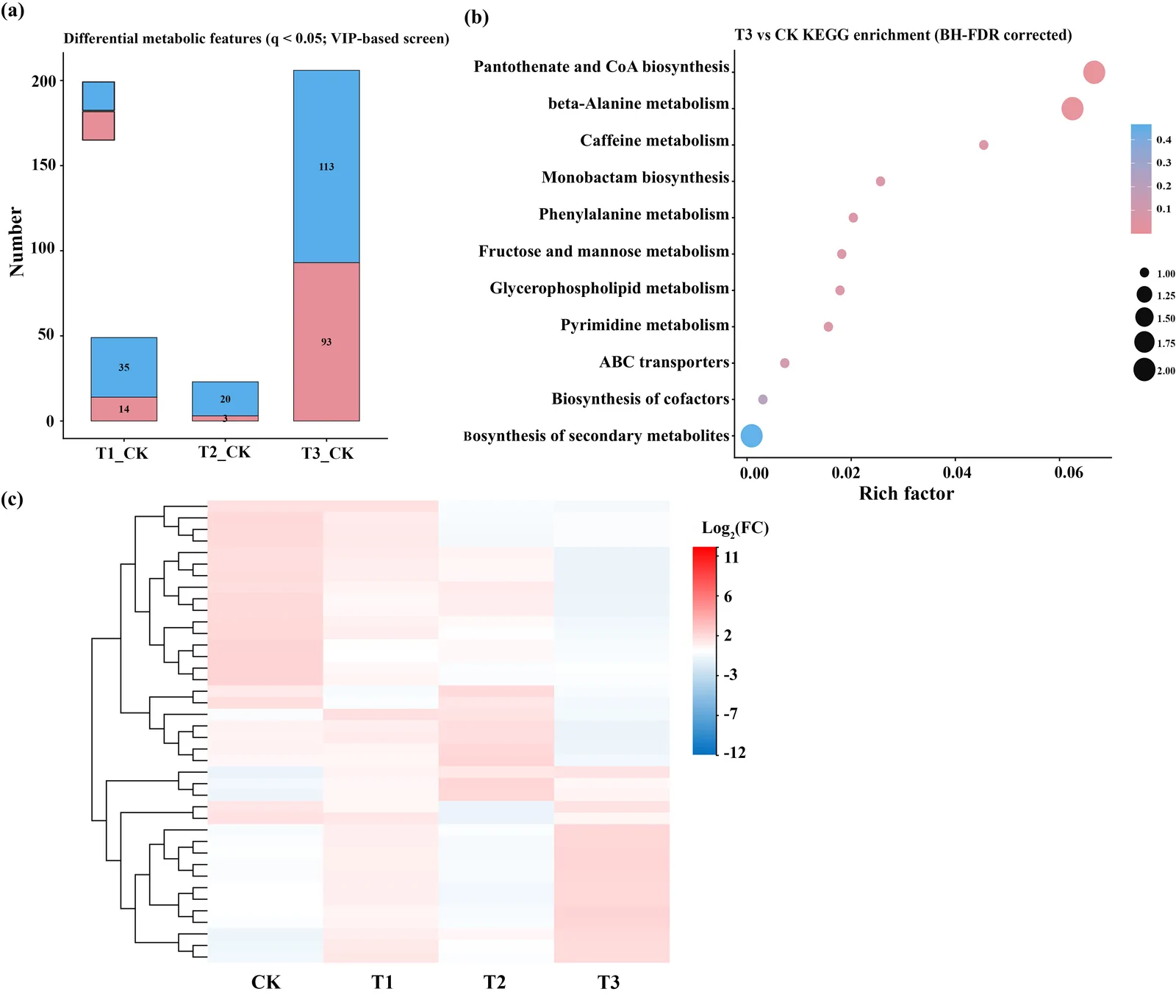
Analysis of differential metabolites in sweet cherry roots under different fertilization treatments. **(A)** The numbers of upregulated and downregulated differential metabolites in the three comparison groups (T1_vs_CK, T2_vs_CK, and T3_vs_CK). X-axis: Comparison group information; Y-axis: Number of differential metabolites. Red represents significantly upregulated metabolites; blue represents significantly downregulated metabolites. **(B)** Metabolic pathway enrichment analysis of differential metabolites in the T3_vs_CK comparison group based on the KEGG database. **(C)** For the union set of differential metabolites across all comparison groups, a clustering heatmap was plotted using their average expression levels in each comparison group. Each row represents one metabolite, and each column represents one biological group. The expression levels were subjected to log_2_ transformation.

Metabolic pathway enrichment analysis was performed based on the KEGG database, with pathways having *P* < 0.05 considered significantly enriched. Due to the limited number of differential metabolites, no significantly enriched pathways were detected in the T1_vs_CK and T2_vs_CK. In contrast, ten metabolic pathways were enriched in the T3_vs_CK: Pantothenate and CoA biosynthesis, beta-Alanine metabolism, Caffeine metabolism, Monobactam biosynthesis, Phenylalanine metabolism, Fructose and mannose metabolism, Glycerophospholipid metabolism, Pyrimidine metabolism, ABC transporters, Biosynthesis of cofactors, and Biosynthesis of secondary metabolites (**Figure 7B**). These pathways are involved in key biogeochemical processes, including nitrogen, phosphorus, and carbon nutrient transformations, as well as microbial substrate transport and energy metabolism. This suggests that the T3 treatment substantially modulated soil material cycling by regulating core metabolic functions. Furthermore, a clustering heatmap was generated based on the union of all differential metabolites across comparison groups, using their average normalized abundance levels in each treatment (**Figure 7C**). The heatmap clearly demonstrated distinct metabolite expression profiles across treatments, visually confirming the regulatory influence of fertilization regimens on soil metabolic characteristics and providing a foundation for elucidating metabolite–microorganism interaction mechanisms.

### 3.10 Correlation analysis between rhizosphere soil microorganisms and metabolites in sweet cherry under fertilization treatments

To investigate the interaction relationship between rhizosphere microorganisms and metabolites, a correlation heatmap was constructed linking dominant microbial taxa with differential metabolites. The T1 was excluded from the analysis due to the presence of only one differential metabolite relative to the control, which was insufficient for robust correlation assessment. As central mediators of material cycling and signal transduction in the rhizosphere microecosystem, metabolites play an essential role in maintaining rhizosphere homeostasis in sweet cherry. Correlation analysis revealed significant associations between key microbial taxa and characteristic metabolites, with certain bacterial genera showing marked regulatory potential in relation to metabolite synthesis or degradation pathways. As shown in **Figure 8A**, in the T2_vs_CK comparison, correlations between bacterial taxa and the metabolites Equol, 9-Deazaguanine, 10-Chloromethyl-11-demethyl-12-oxo-calanolide A, 1-Propenyl 1-(1-propenylthio)propyl disulfide, Vismodegib and 5-Iododeoxyuridine. Genera *Truepera* and *Saccharibacteria* showed strong positive correlations with multiple cyclic and sulfur-containing metabolites, whereas *Gp11*, *Aestuariivirga*, *Gp15* and *Pseudarthrobacter* exhibited significant negative correlations with these compounds. As shown in **Figure 8B**, in the T3_vs_CK comparison, correlations between bacterial taxa and Mannitol, Phosphorylcholine, 4-Hydroxyphenylglycine, Benzthiazide, 3,3’,4,4’-Tetrachloroazoxybenzene and Trimetaphosphoric acid. *Truepera* maintained strong positive associations with Phosphorylcholine and 4-Hydroxyphenylglycine. Notably, *Microvenator* displayed the strongest positive correlation with 3,3’,4,4’-Tetrachloroazoxybenzene (*r* = 0.71). *Gp15* was negatively correlated with mannitol and phosphorylcholine, while showing moderate positive correlations with Benzthiazide and 3,3’,4,4’-Tetrachloroazoxybenzene.

**Figure 8:**
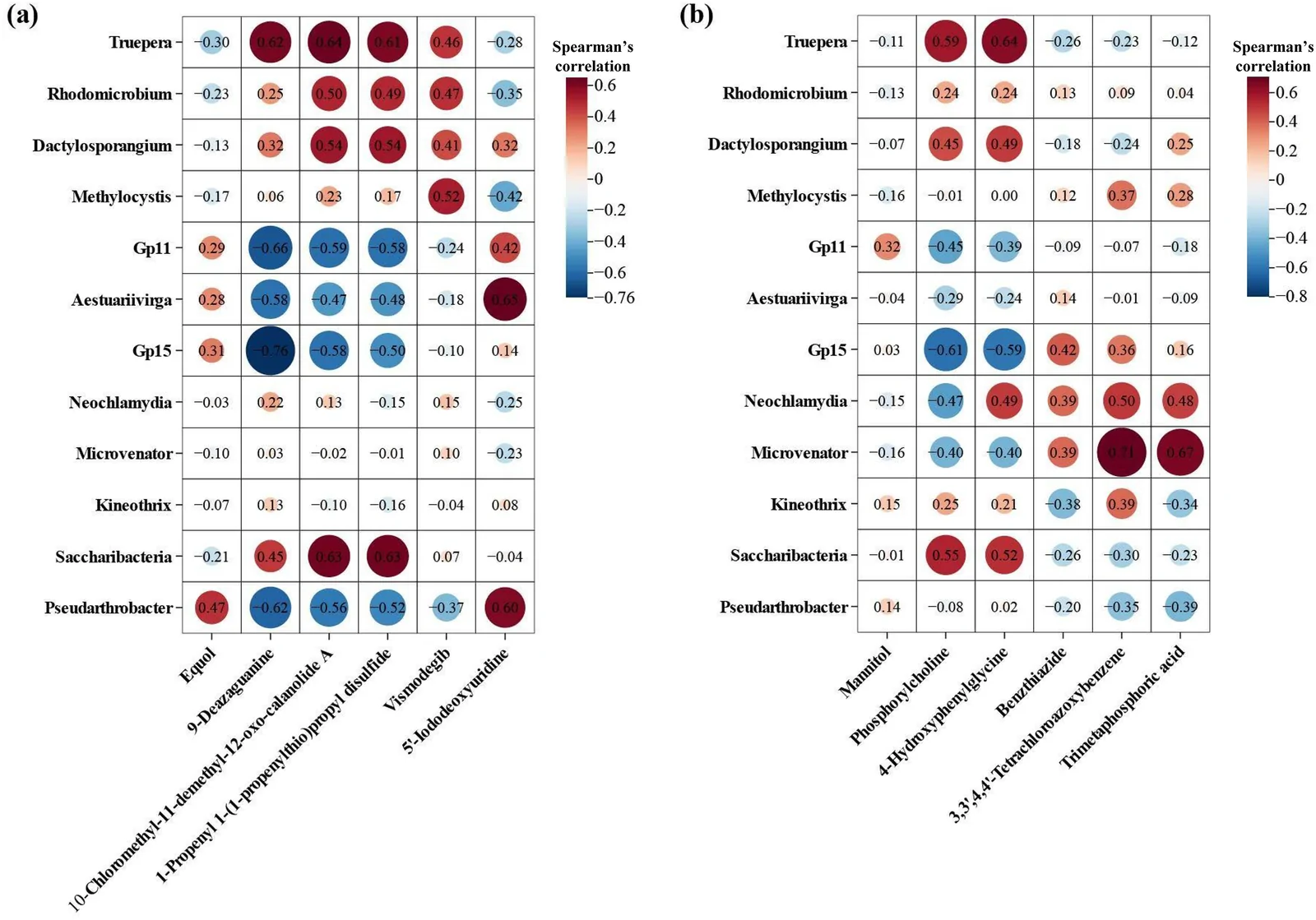
Correlation analysis between rhizosphere soil microorganisms and metabolites in sweet cherry. The bacteria were clearly identified microbial genera and belonged to the top 20 taxa with the highest correlation. Similarly, the metabolites with the highest correlation were selected for heatmap construction. The correlation analyses included two comparisons, namely T2_vs_CK **(A)** and T3_vs_CK **(B)**. Blue indicates a negative correlation, while red indicates a positive correlation.

Path analysis was performed separately for bacterial and fungal communities (**Figure 9**), with path coefficients reflecting descriptive associations among variables measured at a single time point. For the bacterial model (**Figure 9A**), fertilization significantly increased soil physicochemical properties (β = 0.24, *P* = 0.028) and the bacterial community (β = 0.45, *P* = 0.024), but its direct effect on metabolites was not significant (β = 0.19, *P* = 0.337). Soil properties showed a strong negative direct effect on metabolite accumulation (β = −0.65, *P* < 0.001) and a positive effect on bacteria (β = 0.43, *P* = 0.022). The bacterial-to-metabolite path was non-significant (β = −0.11, *P* = 0.682). The model accounted for 27% and 36% of the variance in the bacterial community and metabolites, respectively. In the fungal model (**Figure 9B**), fertilization significantly influenced soil properties (β = 0.24, *P* = 0.041) and the fungal community (β = −0.48, *P* = 0.038), but its direct path to metabolites was non-significant (β = −0.10, *P* = 0.545). Soil properties negatively affected the fungal community (β = −0.86, *P* = 0.005) and positively affected metabolite accumulation (β = 0.36, *P* = 0.031). The fungal-to-metabolite path was not significant (β = −0.50, *P* = 0.344), providing no evidence for fungal mediation of the fertilization effect on metabolites. The model explained 41% and 50% of the variance in fungal community and metabolites, respectively. Collectively, both PLS-PM models pointed to the same conclusion that fertilization acted on metabolites primarily through soil properties, with no statistically supported microbial mediation.

**Figure 9:**
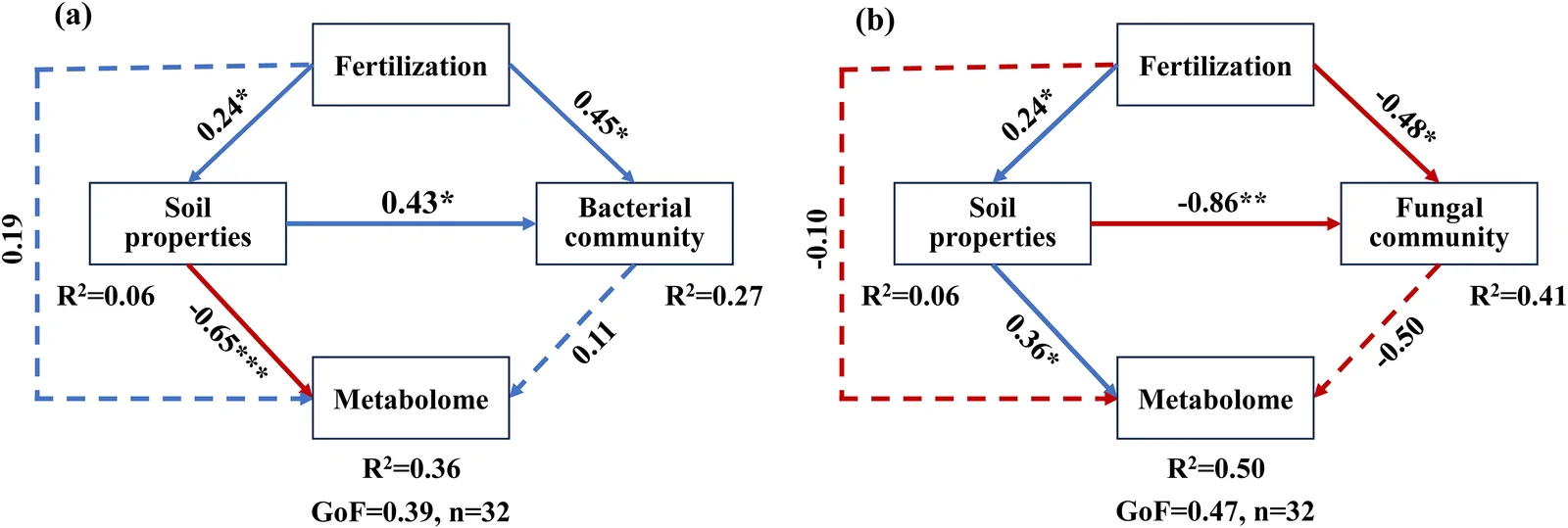
PLS-PM path diagrams illustrating the cascading causal pathway of fertilization (CK, T1, T2, T3) altering soil physicochemical properties, dominant microbial communities and soil metabolome. **(A)** Bacterial community model (GoF = 0.39, n = 32); **(B)** Fungal community model (GoF = 0.47, n = 32). Solid arrows denote significant paths and dashed arrows denote non-significant paths; blue and red colors denote positive and negative coefficients, respectively. Arrow-labeled figures are standardized path coefficients (β), boxed figures are R^2^. Path coefficients and significance levels are labeled beside each arrow (*P < 0.05; **P < 0.01; ***P < 0.001; 500 bootstrap resamples).

## 4 Discussion

### 4.1 Effects of combined fertilization regimes on soil properties, microbial communities and rhizosphere metabolites

As a core agricultural practice for regulating soil nutrient status, fertilization induces perturbations in the rhizosphere microecosystem that are ultimately reflected in alterations to microbial community structure and metabolic profiles (Yuan et al., 2022). Using sweet cherry rhizosphere soil as the study system, this research systematically evaluated the effects of different organic substrate combinations supplemented with EM inoculant on soil physicochemical characteristics, soil microbial communities and metabolites, and elucidated their correlation patterns. These findings provide essential data support for understanding the intrinsic mechanisms underlying fertilization-mediated regulation of the rhizosphere microecosystem.

All fertilization treatments significantly increased soil organic matter and available N, P, K relative to CK. Organic manure supplies abundant exogenous carbon and mineral nutrients, while porous biochar reduces nutrient leaching and EM accelerates organic matter decomposition and nutrient activation (Troy et al., 2014; Ardian et al., 2025). Unexpectedly, T3 outperformed T1 and T2 in improving soil fertility, which could be attributed to the fact that the biochar combined with beneficial microbes can effectively mobilize indigenous soil nutrients without extra manure input. Soil pH gradually declined from CK to T3. Organic nitrogen nitrification and organic acid accumulation during organic material mineralization are the main causes of soil acidification (Du et al., 2024). The lowest pH in T3 was probably caused by enhanced microbial metabolism driven by EM and biochar. Importantly, all pH values stayed within the optimum range for sweet cherry root growth. In summary, the T3 was the optimal amendment to improve physicochemical properties of greenhouse sweet cherry orchard soil.

Petal plot analysis showed that the number of shared bacterial ASVs across the four treatments (3,876) far exceeded the corresponding number for fungi (152), indicating greater stability of core bacterial taxa in the sweet cherry rhizosphere, whereas the fungal community harbors a larger proportion of treatment-specific, unique taxa. This disparity, reflects the greater stability of the bacterial core microbiome under fertilization. All three amended treatments received the same EM inoculant, which consisted predominantly of bacterial taxa (photosynthetic bacteria, lactic acid bacteria, and actinomycetes), and the manure inputs in T1 and T2 further supplemented bacterial inoculator. Therefore, the abundant shared bacterial ASVs likely arose, at least in part, from the applied amendments rather than representing an intrinsic core microbiome of the sweet cherry rhizosphere. Fungal communities, by contrast, showed substantially fewer shared ASVs (152), a pattern consistent with the typically stronger host filtering and dispersal limitation of fungi in perennial crop systems, and also with the fact that none of the organic inputs exerted strong fungal-selective effects. These observations suggest that the bacterial core inferred from shared ASVs may have been artificially inflated by experimental design, whereas the fungal community provided a more conservative estimate of the indigenous rhizosphere assemblage. Notably, T3 contributed the largest unique bacterial set (55 ASVs), whereas T2 contributed the largest unique fungal set (23 ASVs), implying that T3 preferentially recruited bacterial taxa whereas the T2 preferentially recruited fungal taxa. The PCoA ordinations indicated statistically significant overall differences in microbial community composition across treatments (PERMANOVA and ANOSIM, both *P* < 0.001), yet the separation among group centroids was relatively limited, as reflected by the extensive overlap of 95% confidence ellipses in the reduced ordination space. Betadisper tests confirmed that within-group dispersion did not differ significantly among treatments for either bacteria or fungi, ruling out heterogeneity in multivariate spread as a confounding factor underlying the significant PERMANOVA and ANOSIM outcomes. Rather, these results suggest a genuine but modest centroid shift in response to fertilization. The effect sizes obtained were low, and the marked ellipse overlap further underscores that, despite statistical significance, the observed compositional differences were not associated with clear segregation of treatment groups in the ordination plots. Thus, the inferred fertilization effects should be viewed as statistically discernible yet ecologically moderate, indicating shifts in community composition that fall short of strong group-level differentiation. Given the modest magnitude of separation and the inherent sensitivity of distance-based tests to sample size and data structure, future investigations with larger per-treatment replication will be necessary to disentangle the relative contributions of centroid location and dispersion to beta-diversity patterns under fertilization regimes (Warton et al., 2012).

Microbial community diversity and composition constitute the foundation of ecological functionality and are highly sensitive to environmental changes (Li et al., 2024a; Yang et al., 2017). In terms of community composition, the dominant bacterial phyla across all treatments included Pseudomonadota, Actinomycetota, and Acidobacteriota, which is consistent with the bacterial diversity associated with lichens in Mt. Yunmeng (Li et al., 2023). The T1 significantly increased the relative abundances of Bacteroidota and Pseudomonadota. These phyla predominantly consist of heterotrophic microorganisms capable of secreting extracellular enzymes such as cellulase and protease, enabling efficient decomposition and utilization of the abundant organic nutrients present in sheep manure. The enrichment of these taxa likely represents an adaptive response to elevated soil organic carbon and nitrogen levels following fertilization (Larsbrink and McKee, 2020; Naziębło and Dobrzyński, 2025; Martin et al., 2025). The T2 treatment promoted the accumulation of Actinomycetota and Acidobacteriota. Previous studies have shown that soil organic matter, available nitrogen, and available phosphorus are key drivers of Actinomycetota enrichment (Song et al., 2025), suggesting that the T2 treatment enhanced soil nutrient availability. Within the fungal community, Ascomycota was the predominant phylum, accounting for over 90% of total fungal sequences. All fertilization treatments significantly increased the abundance of Ascomycota while reducing those of Mortierellomycota and Basidiomycota, potentially enhancing organic matter decomposition and promoting soil nutrient cycling (Bai et al., 2024). *Ascomycota* includes saprophytic fungi known for their strong adaptability to soils with high carbon-to-nitrogen ratios (Liu et al., 2020; Sharma et al., 2018). At the genus level, the dominant bacterial *Stenotrophobacter* and *Sphingomonas* mostly possess plant growth-promoting, phosphate-solubilizing, and nitrogen-fixing functions, which may be closely related to the improved soil nutrient availability after fertilization (Asaf et al., 2020; Sato et al., 2023). In dominant fungi, T3 significantly enhanced organic matter decomposition by enriching saprophytic/generalist fungi (*Fusarium*, *Cladosporium*, *Dactylonectria*), accelerating nutrient cycling, but the simultaneous enrichment of *Dactylonectria* and *Fusarium* warrants caution, as both include plant-pathogenic species that have been associated with root rot and replant disease in perennial fruit crops (Probst et al., 2019; Silva et al., 2020). Long-term field trials are required to assess whether the Biochar+EM treatment may inadvertently elevate soilborne disease pressure under greenhouse monoculture conditions.

As central mediators of material cycling and signal transduction in the soil microecosystem, changes in the composition and concentration of rhizosphere metabolites directly reflect the intensity and direction of soil metabolic activities (Massalha et al., 2017). Using LC-MS technology, this study identified 355 known metabolites, primarily enriched in 13 metabolic pathways, including xenobiotic biodegradation and metabolism, amino acid metabolism, and carbohydrate metabolism. These pathways encompass core processes of soil nutrient transformation and microbial energy metabolism, underscoring the high functional complexity of the sweet cherry rhizosphere. Differential metabolite screening revealed marked variation in the extent of metabolic perturbation across treatments. The T3 treatment exhibited the highest number of differential metabolites compared to the control (206 metabolites), followed by T2 with 23 differential metabolites. These findings suggest that the T3 exerted the strongest influence on the soil metabolic system, whereas the T1 treatment had the least impact. It is hypothesized that the gradual release of organic nutrients from sheep manure resulted in milder disturbances to the native soil metabolic equilibrium (Haider et al., 2024). In contrast, the combined application of biochar and microbial inoculant may more strongly regulate metabolite profiles by modifying soil pore structure and stimulating microbial metabolic activity (Nie et al., 2024). KEGG enrichment analysis indicated that differential metabolites in the T3 were significantly enriched in ten pathways, including pyrimidine metabolism, phenylalanine metabolism, and glycerophospholipid metabolism. Pyrimidine metabolism plays a critical role in microbial nucleic acid synthesis and energy transfer (Stasolla et al., 2003); its enrichment suggests enhanced microbial proliferation and metabolic activity under T3. Phenylalanine metabolism is closely linked to the biosynthesis of secondary metabolites such as flavonoids and phenolic compounds, which function as signaling molecules regulating plant–microbe interactions (Pascual et al., 2016; Tzin and Galili, 2010; Ketehouli et al., 2025). This pathway may represent a key mechanism through which the T3 treatment modulates functional traits in the rhizosphere microecosystem. Glycerophospholipid metabolism is involved in cell membrane synthesis and repair (Janda et al., 2013); its upregulation implies that fertilization may alter microbial membrane composition to facilitate adaptation to changing soil conditions.

Complex interactions exist between rhizosphere microorganisms and metabolites: microorganisms can synthesize or degrade metabolites through metabolic activity, while metabolites can shape microbial community structure by selectively enriching or inhibiting specific taxa (Mishra et al., 2022). Correlation analysis revealed treatment-specific patterns of microbe–metabolite associations across fertilization regimes, with distinct bacterial genera showing significant negative or positive correlations with selected differential metabolites in the T2_vs_CK and T3_vs_CK. These differential metabolites primarily belong to amino acid derivatives, organic acids, and nucleotide-related compounds, which may reflect treatment-induced shifts in microbial metabolic activity and organic matter turnover (Kallenbach et al., 2016). Additionally, differential tolerance among microbial taxa to these metabolites may contribute to the distinct correlation patterns observed under different fertilization regimes (Cheng et al., 2022). Metabolite-microbe interactions imply potential metabolic cooperation or competition mechanisms, which together construct the complex metabolic landscape of the rhizosphere (Jiang et al., 2024).

In both path models, the opposing associations of bacterial and fungal communities with soil physicochemical properties match the copiotroph-oligotroph pattern in which copiotroph bacteria are favored and fungi are suppressed under nutrient-richer, lower-pH conditions (Yao et al., 2014; Zhou et al., 2015). The path analysis detected no significant associations between microbial blocks and the metabolome, while soil physicochemical properties were directly associated with the metabolome. Metabolite pools can respond on shorter timescales than microbial community turnover, and taxa-specific metabolic signals may be diluted under nutrient-surplus conditions (Allison and Martiny, 2008). Intensive fertilization for protected sweet cherry appears to coincide with a shift in soil microbial dominance from fungi to bacteria, which may accelerate nutrient cycling but could compromise fungal contributions to organic matter decomposition, mycorrhizal symbiosis and aggregate stability (Allison and Martiny, 2008). Accordingly, we recommend sustainable fertilization management for protected sweet cherry orchards to maintain organic amendments (e.g., compost and biochar) and prevent excessive soil acidification, so as to conserve fungal communities and soil multifunctionality.

### 4.2 Limitations of the experimental design

Three limitations of the present experimental design should be acknowledged. First, the greenhouse experiment employed a composite-treatment design (CK, T1, T2, and T3), in which each amended group received a combination of livestock manure (sheep or chicken), biochar, and EM inoculant. No single-factor control groups (e.g., sheep manure alone, chicken manure alone, biochar alone, or EM inoculant alone) were included. As a result, the independent main effects and potential interactive effects among manure source, biochar, and EM inoculant on rhizosphere microbial communities and metabolite profiles cannot be separately assessed from the current dataset. The observed inter-treatment differences (e.g., T3 vs. CK) thus represent the integrated response to the composite amendment regime, rather than the contribution of any individual component. Future factorial or single-factor control designs, incorporating each amendment separately under otherwise identical conditions, would be required to disentangle the individual and interactive contributions of these amendments and to identify the primary drivers of the microbial community shifts and metabolite accumulation observed here. Second, while we characterized the pre-treatment soil physicochemical properties for all four greenhouses and included these baselines as covariates in the linear mixed-effects models, we did not collect corresponding baseline data for microbial communities or metabolomic profiles. Consequently, the observed differences among treatments represent absolute values at the end of the experiment, rather than true net changes attributable to the amendments within each plot. Finally, the relatively small sample size (n = 32) constrains the statistical power and may compromise the stability and generalizability of the results. This concern is particularly relevant for the metabolite block, where 16 indicators were derived from the same 32 observations, yielding an indicator-to-sample ratio substantially below the conventional 10:1 rule of thumb. We therefore treat the path coefficients from this block as exploratory rather than confirmatory. Future research will conduct multi-site and multi-season sampling combined with high-frequency metabolomic monitoring, to further unravel the temporal decoupling mechanism between microbial succession and metabolite turnover.

## 5 Conclusion

In summary, different fertilization regimes significantly altered soil physicochemical properties, microbial community structure, and rhizosphere metabolic profiles of greenhouse sweet cherry. All fertilization treatments effectively increased soil nutrient availability and soil organic matter while slightly reducing soil pH. Although microbial alpha diversity indices showed no significant differences among treatments, fertilization markedly reshaped microbial community composition, structure, and co-occurrence patterns. The T3 induced the most pronounced changes in soil metabolic characteristics and differential metabolite accumulation. PLS-PM further demonstrated that fertilization affected soil metabolite accumulation predominantly through soil physicochemical properties in both bacterial and fungal models, while the originally hypothesized microbial-mediation pathway was not statistically supported. Overall, within this experimental design, the combined application of biochar and EM inoculants was the most suitable fertilization strategy, effectively optimizing rhizosphere microecological conditions of greenhouse sweet cherry orchard soil. However, its long-term suitability remains uncertain, because T3 enriched root-rot-associated genera (*Fusarium*, *Dactylonectria*) that may elevate soilborne disease risk. Long-term application therefore warrants caution, and field validation under continuous monoculture is required.

## Data Availability

Raw data have been deposited to National Center for Biotechnology Information (NCBI) under the BioProject number PRJNA1517584 and PRJNA1517571. Source data have been deposited to the EMBL-EBI MetaboLights database with the identifier MTBLS15458.

## Supplementary material

Supplementary Figure 1 Geographic location and digital elevation model (DEM) of the study site in Wenshu Village, Jiayuguan City, Gansu Province, China. The inset map illustrates the geographical location of Jiayuguan City within Gansu Province.

Supplementary Figure 2 Alpha diversity indices of bacterial **(A)** and fungal **(B)** communities under different treatments. Chao1, Observed, Shannon, and Simpson indices are presented. Differences among treatments were evaluated using the Kruskal-Wallis test, with the results shown in **Supplementary Table S2**.

Supplementary Figure 3 Rarefaction curves for bacteria **(A, B)** and fungi **(C,D)**.

Supplementary Figure 4 The species composition of GraPhlAn and the LEfSe results in the bacterial category. **(A)** The GraPhlAn species composition plot is primarily used for the holistic visualization of species composition across different taxonomic levels in samples. It employs distinct colors to differentiate individual taxonomic units, uses node sizes to reflect species abundances at each taxonomic level, and applies the color intensity of the outer circular heatmap to characterize species abundances across groups, which can be utilized to identify dominant microbial taxa. **(B)** Results of LEfSe analysis showing bacterial taxa that differed significantly across the three treatments.

Supplementary Figure 5 The species composition of GraPhlAn and the LEfSe results in the fungal category. **(A)** The GraPhlAn species composition plot is primarily used for the holistic visualization of species composition across different taxonomic levels in samples. It employs distinct colors to differentiate individual taxonomic units, uses node sizes to reflect species abundances at each taxonomic level, and applies the color intensity of the outer circular heatmap to characterize species abundances across groups, which can be utilized to identify dominant microbial taxa. **(B)** Results of LEfSe analysis showing fungal taxa that differed significantly across the three treatments.

Supplementary Figure 6 Bacterial and fungal co-occurrence networks under each treatment.

Supplementary Figure 7 Non-metric multidimensional scaling (NMDS) ordination of bacterial **(A)** and fungal **(B)** communities based on Bray-Curtis dissimilarity.

Supplementary Figure 8 OPLS-DA R^2^Y/Q^2^ validation index.

Supplementary Table 1 Baseline soil physicochemical characteristics of four greenhouses before treatment application.

Supplementary Table 2 Goodness-of-fit and reliability indices of the partial least squares path models (PLS-PM) for bacterial and fungal community pathways.

Supplementary Table 3 Differences in alpha diversity indices among treatments were tested using the Kruskal-Wallis test.

Supplementary Table 4 Overview of QC and annotation for metabolomics data.

Supplementary Table 5 Annotation and differential expression information of annotated metabolites.
